# Enzymatic Synthesis of RNAs Capped with Nucleotide Analogues Reveals the Molecular Basis for Substrate Selectivity of RNA Capping Enzyme: Impacts on RNA Metabolism

**DOI:** 10.1371/journal.pone.0075310

**Published:** 2013-09-25

**Authors:** Moheshwarnath Issur, Isabelle Bougie, Simon Despins, Martin Bisaillon

**Affiliations:** Département de Biochimie, Faculté de Médecine et des Sciences de la Santé, Université de Sherbrooke, Sherbrooke, Quebec, Canada; University of Surrey, United Kingdom

## Abstract

RNA cap binding proteins have evolved to specifically bind to the N7-methyl guanosine cap structure found at the 5’ ends of eukaryotic mRNAs. The specificity of RNA capping enzymes towards GTP for the synthesis of this structure is therefore crucial for mRNA metabolism. The fact that ribavirin triphosphate was described as a substrate of a viral RNA capping enzyme, raised the possibility that RNAs capped with nucleotide analogues could be generated *in cellulo*. Owing to the fact that this prospect potentially has wide pharmacological implications, we decided to investigate whether the active site of the model *Paramecium* *bursaria* *Chlorella virus-1* RNA capping enzyme was flexible enough to accommodate various purine analogues. Using this approach, we identified several key structural determinants at each step of the RNA capping reaction and generated RNAs harboring various different cap analogues. Moreover, we monitored the binding affinity of these novel capped RNAs to the eIF4E protein and evaluated their translational properties *in cellulo*. Overall, this study establishes a molecular rationale for the specific selection of GTP over other NTPs by RNA capping enzyme It also demonstrates that RNAs can be enzymatically capped with certain purine nucleotide analogs, and it also describes the impacts of modified RNA caps on specific steps involved in mRNA metabolism. For instance, our results indicate that the N7-methyl group of the classical N7-methyl guanosine cap is not always indispensable for binding to eIF4E and subsequently for translation when compensatory modifications are present on the capped residue. Overall, these findings have important implications for our understanding of the molecular determinants involved in both RNA capping and RNA metabolism.

## Introduction

The addition of a 5’ cap structure to RNA transcripts synthesized by RNA polymerase II is fundamental to eukaryotic gene expression [1]. The N7-methyl guanosine cap in mRNAs is essential for their stability, maturation, transport and translation. The presence of the methyl group on the guanosine residue, which confers a positive charge to the cap structure, allows its specific recognition by various specialized proteins. The cap structure fulfills many roles that ultimately lead to mRNA translation. In the nucleus for instance, the cap structure of pre-mRNA is recognized by the cap binding proteins (CBP20 et CBP80) [2]. This cap binding complex (CBC) protects mRNA from degradation and assists RNA transport from the nucleus to the cytoplasm. Once in the cytoplasm, ribosomes must be recruited for efficient translation of mRNAs into proteins. The eukaryotic translation initiation factor 4E (eIF4E) specifically binds to the RNA cap structure [3]. This association is mediated by two aromatic residues of the eIF4E protein; the mRNA binding is further stabilized by specific hydrogen bonds between the positive charge of the 7-methylguanosine and an acidic residue (reviewed in [4]). Upon cap binding, eIF4E assembles with eIF4G (a scaffold protein) and eIF4A (an RNA helicase) into the eIF4F complex [5]. The scaffolding protein eIF4G recruits the small 40S ribosomal subunit through the eIF3 complex [6]. The translation initiation complex then scans the mRNA for the start codon before recruiting the larger subunit of the ribosome, which proceeds to the open reading frame translation [4]. Taken together, the roles fulfilled by the RNA cap structure are crucial for RNA stability and translation.

RNA cap synthesis occurs in 3 consecutive enzymatic steps. An RNA triphosphatase (RTase) first cleaves the 5’ terminal phosphate of the RNA molecule, to form a diphosphorylated end. An RNA capping enzyme, or RNA guanylyltransferase (GTase), transfers a GMP moiety onto the diphosphorylated end, followed by an RNA (guanine-N7) methyltransferase (MTase) which adds a methyl group at the N7 position to form the classical RNA cap structure [7]. In cells, the RNA cap structure is bound by several RNA cap binding proteins which have evolved to recognize a N7-methylated guanosine base. Therefore the specificity of the GTase for GTP is of paramount importance to ensure the formation of a functional RNA cap structure.

**Figure 1:**
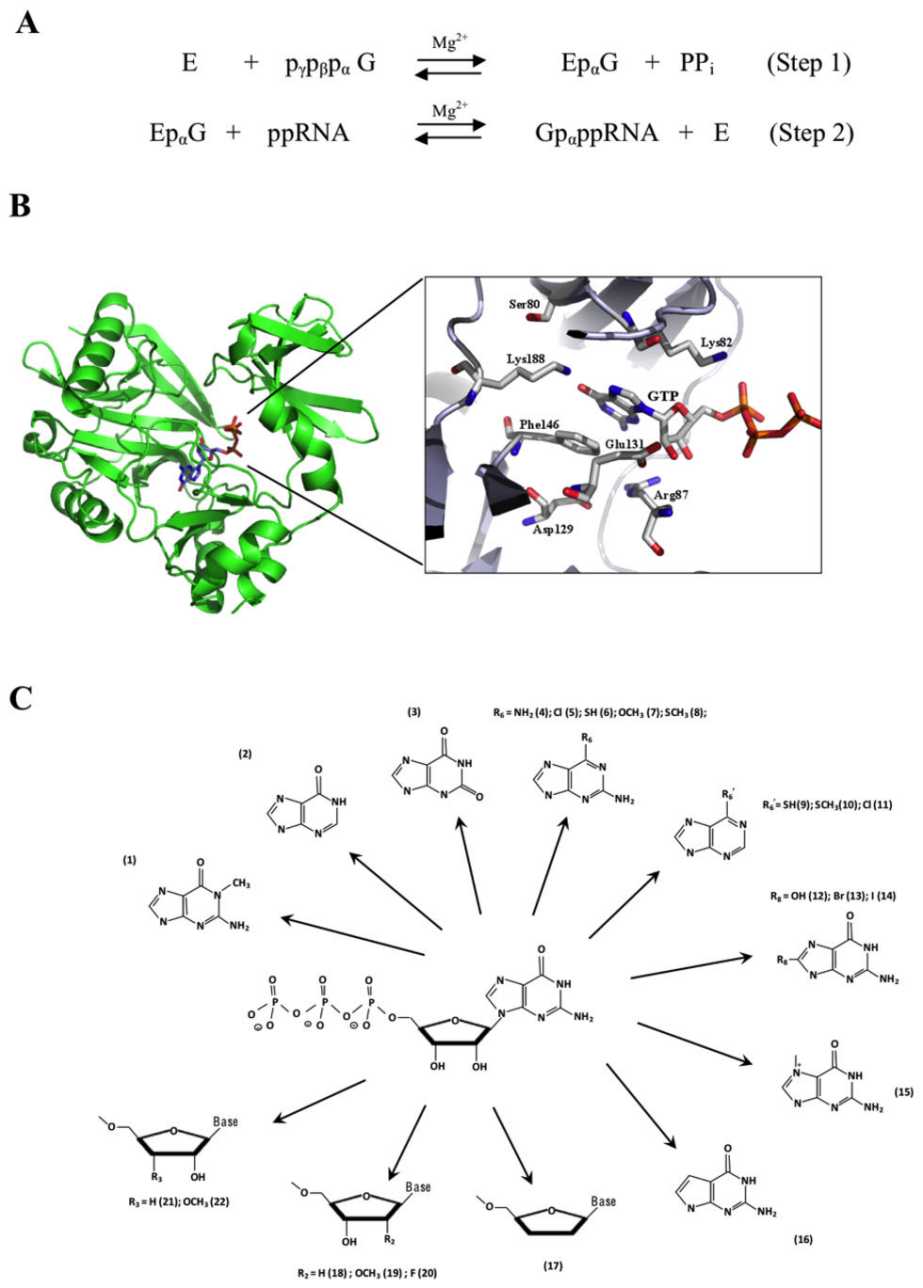
The RNA capping mechanism and the nucleotide analogues tested (A) The two-step RNA guanylyltransferase reaction. (B) The GTP binding site of the PBCV-1 GTase (PDB 1CKN). Residues shown are those interacting with the base and the sugar moiety. (C) Nucleotide analogues used in this study.

GTases first hydrolyze GTP to form a covalent enzyme*-*(*lysyl-N*)-GMP intermediate prior to transferring the GMP moiety onto an acceptor RNA (Figure 1A). GTases share the same mechanistic profile as well as six co-linear conserved motifs, including the catalytic KxDG motif, with ATP dependent ligases (Figure S1) [8]. Crystal structures of the *Paramecium* *bursaria* *Chlorella virus-1* (PBCV-1) GTase and T7 DNA ligase bound to GTP and ATP respectively have revealed a common tertiary fold in which these conserved motifs are brought together at the active site of the enzyme [9]. While the modulation of the GTP specificity of GTases by the rational design of point mutations remain to be achieved, the finding that ribavirin triphosphate could substitute GTP and be incorporated at the 5’ end of an RNA by a viral GTase has shed some novel insights into substrate recognition by GTases [10]. Owing to the potential pharmacological value of the possibility of generating RNA cap analogues *in vivo*, we decided to probe into the structural flexibility of the GTP binding site of a model GTase by measuring the effectiveness of various purine analogues to sustain each step of the RNA capping reaction. Although synthetic nucleotide analogues have been extensively studied with regards to RNA stability and translation [11-15], few studies have aimed to probe into the capping machinery itself to understand the underlying interactions involved in GTP binding for RNA cap synthesis. In order to understand the modulation of substrate specificity in RNA capping enzymes, this report aims to identify the essential interactions at each step of the RNA capping pathway. Owing to its extensive biochemical and structural characterization, the PBCV-1 GTase was the ideal tool to delve further into the mechanism of RNA capping [9,16]. RNAs harboring several novel cap structures bearing unusual substitutions on the base moiety were thus generated enzymatically. The effects of these modifications on RNA metabolism were then investigated.

## Methods

### Expression and purification of the relevant proteins

Recombinant His-tagged PBCV-1 GTase, PBCV-1 RTase, *S. cerevisiae* MTase, human MTase and murine eIF4E proteins were expressed in bacteria as described before [17-21].

### Preparation and purification of RNA substrates

RNA substrates were synthesized with the MAXIscript kit (Ambion) using T7 RNA polymerase. For the evaluation of RNA capping efficiencies, a 50 nt long RNA was synthesized using a modified version of the T7 promoter that will allow the initiation of the transcription with a single guanosine residue (5'-TAATACGACTCACTATA↓G A_49_-3', where ↓ indicates the initiation start site). *In cellulo* translation assays, were done with the *firefly* luciferase RNA bearing a 60 nt long polyA tail at its 3’ end, which was synthesized from an HpaI digested *plucA*_*60*_ plasmid (a generous gift from Dr. Rhoads, Louisiana State University). Following transcription, the RNA molecules were purified on denaturing 8M UREA-PAGE and visualized by ultraviolet shadowing. The corresponding band was excised and then eluted from the gel by an overnight incubation in 0.1% SDS and 0.5 M ammonium acetate. The RNA was then precipitated with ethanol and quantified by spectrophotometry at 260 nm.

### Nucleotide Analogues

All nucleotide analogues used in this study were purchased from Jena Biosciences (Germany) and TriLink Biotechnologies (USA).

### First step RNA guanylyltransferase reaction

The first step of the GTase reaction was carried out by incubating the purified PBCV-1 GTase (10 µM) with the appropriate substrates (GTP or nucleotide analogues) in a buffer containing 50 mM Tris-HCl, pH 7.5, 5 mM DTT, 5 mM MgCl_2_ and 1.5 µg/ml of inorganic yeast pyrophosphatase (Roche) for 1hr at 30°C.

### Inhibition assay and determination of the IC_50_

Inhibition of the first step of the GTase reaction was evaluated by carrying out the standard first step GTase reaction in the presence of 0.2 pmol of [α-^32^P] GTP and 2 mM of either unlabelled GTP or each unlabelled nucleotide analogue separately. The IC_50_ of nucleotide analogues was determined by carrying out the standard first step reaction in the presence of 0.2 pmol of [α-^32^P] GTP and increasing concentrations of up to 2 mM of each nucleotide analogue. The reactions were stopped by the addition of EDTA to 10 mM and SDS to 1% and analyzed by electrophoresis through a 12.5% polyacrylamide gel containing 0.1% SDS. The radiolabelled proteins were visualized by autoradiography of the gel. Radiolabelled covalent complex formation was quantified by scanning the gel with a PhosphorImager (Amersham Biosciences).

### Evaluation of the formation of the covalent intermediate

The standard first step reaction was carried out in the presence of each nucleotide analogue (2 mM) in the presence or absence of potassium pyrophosphate (1 mM). The reactions were resolved on SDS-PAGE followed by Coomassie blue staining.

### *In vitro* RNA capping reaction

RNA capping was performed by incubating the purified PBCV-1 GTase (10 µM) and PBCV-1 RTase (5 µM), either GTP (2 mM) or a nucleotide analogue (2 mM) with the appropriate RNA in a buffer containing 50 mM Tris-HCl, pH 7.5, 5 mM DTT, 5 mM MgCl_2_ and RnaseOut (1 unit) (Invitrogen).

### Transfer of nucleotide analogues to RNA

The transfer of GTP or of a nucleotide analogue onto RNA was assayed by performing the *in vitro* RNA capping reaction in the presence of an internally labelled 50 nt long RNA substrate (10 pmol). The RNA was extracted with phenol/chloroform and recovered by ethanol precipitation before being analyzed on a denaturing Urea-PAGE. Autoradiography of the gel with a PhosphorImager enabled discrimination between a cap and uncapped RNA. Alternatively, a 50 nt long RNA molecule with a 5’ α-labelled guanosine residue (5’-pp^32^p RNA-3’) was used. To evaluate capping efficiency, the GTase reaction mixture was heated to 95°C for 3 minutes before being adjusted to 50 mM sodium acetate (pH 5.2) and digested with nuclease P1 (5µg) and alkaline phosphatase (1 unit) at 37°C for 1 hr. The products were then analyzed by thin layer chromatography (TLC) on a polyethyleneimine-cellulose plate developed with 0.5 M LiCl and 1 M formic acid, following which, the extent of cap formation was measured by scanning the TLC with a PhosphorImager. Cap formation is inferred from the presence of unhydrolyzable 5’-5’ triphosphate bridge (Npp^32^pG) on the TLCs.

### Cell culture

Human embryonic kidney cells (HEK293) were maintained in Dulbecco’s modified Eagle’s medium supplemented with 10% fetal bovine serum.

### Transfection

The day before transfection 600,000 cells/well were distributed in a 6-well plate. LucA_60_ RNA (5 µg) was transfected using the Qiagen Transmessenger kit according to the manufacturer’s protocol for 3 hr. Each RNA species was transfected twice per experiment. For each RNA species transfected, the cells in one of the wells were washed with PBS and harvested, while in the other, the cells were incubated at 37°C in pre-warmed complete medium for 6 hr, following which, the cells were washed and harvested.

### Determination of translation efficiency

The harvested cells were split into 2 tubes. Cells in one of the tubes were lysed in Luciferase Cell Culture Lysis Reagent (Promega) and luciferase activity was measured according to the manufacturer’s protocol (Promega). Total protein concentration of cell extracts was determined by the Bio-Rad dye binding method, using bovine serum albumin as the standard. Relative luciferase units (RLU) reads were rationalized onto the total protein concentration in the extracts and the capping efficiency of each nucleotide analogue tested. Translation was assumed to be due to capped transcripts only. Luciferase readings were corrected to RLU/ug of capped LucA_60_ RNA only. The proportion of capped RNA was evaluated from the calculation of the capping efficiency. The translation efficiency was determined by normalizing the data relative to the natural N7-methyl guanosine cap lucA_60_ RNA.

### Determination of RNA levels post-transfection

Harvested cells were lysed in Qiazol and RNA was extracted according to the manufacturer’s protocol (Qiagen). Real-time quantitative PCR analysis using total RNA extracts was performed as described previously [22]. Briefly, 1 µg of total RNA was treated with Promega DNase RQ1 and reverse transcribed using Qiagen Omniscript RT. cDNAs were diluted 20-fold and analyzed on an Eppendorf Realplex PCR instrument using PerfeCTa^TM^ SYBR Green Supermix kit (Quanta Biosciences). LucA_60_ RNA levels were quantified relative to the GAPDH RNA using the ΔΔ^Ct^ method as previously described [22]. RNA levels were determined by comparing relative lucA_60_ RNA levels 6 hr post-transfection relative to 0 hr post-transfection. The data presented has been normalized relative to the stability of the N7-methyl guanosine capped lucA_60_ RNA.

### *In vitro* translation assay

*In vitro* translation assays were performed by incubating N7-methyl guanosine cap lucA_60_ RNA (1µg) with the Rabbit Reticulocyte Lysate (Promega) for 10 minutes at 30°C.

### *In vitro* RNA binding assay to eIF4E

The binding of RNAs harboring modified RNA cap structures to the eIF4E protein was evaluated by fluorescence spectroscopy as described previously [23]. Briefly, excitation was performed at a wavelength of 290 nm fluorescence using a Hitachi F-2500 fluorescence spectrophotometer. Background emission was eliminated by subtracting the signal from the buffer containing the RNA substrate. The extent to which the RNA binds to purified eIF4E protein was determined by monitoring the fluorescence emission of a fixed concentration of proteins with and without the appropriate RNA (2 µM). All the values were rationalized relative to the capping efficiency of each nucleotide analogue (Table 1) and are presented relative to the fluorescence quenching observed when eIF4E binds the natural N7-methyl guanosine cap.

**Table 1:** RNA capping reaction with nucleotide analogues.

**Analogues**	**IC50 for E-GMP formation (mM**)**^a^*1*^*st*^*step***	**Formation of a covalent complex^b^*1*^*st*^*step***	**Relative efficiency of RNA capping^c^*1*^*st*^*and2*^*nd*^*steps*^*e*^**
GTP	0.10	+	1.0
ATP	>2.0	-	-
A_1_ N1-Me GTP	0.08	+	1.1
A_2_ ITP	0.34	+	1.3
A_3_ XTP	>2.0	-	-
A_4_ 2-Amino ATP	>2.0	-	-
A_5_ 2-Amino-6 Cl purine RTP^d^	0.16	+	0.08
A_6_ 6-thio GTP	0.22	+	0.16
A_7_ O6-Me GTP	0.15	+	0.48
A_8_ 6-Me thio GTP	0.43	+	0.07
A_9_ 6- thio ITP	1.7	+	-
A_10_ 6-Me thio ITP	1.2	+	-
A_11_ 6-Cl purine RTP^d^	>2.0	-	-
A_12_ 8-Oxo GTP	>2.0	-	-
A_13_ 8-Bromo GTP	1.5	+	0.40
A_14_ 8-Iodo GTP	0.42	+	1.0
A_15_ N7-Me GTP	>2.0	-	-
A_16_ 7-deaza GTP	>2.0	-	-
A_17_ ddGTP	>2.0	-	-
A_18_ 2’dGTP	>2.0	-	-
A_19_ 2’O-Me GTP	>2.0	-	-
A_20_ 2’ F-2’ dGTP	1.4	+	0.16
A_21_ 3’dGTP	0.15	+	0.15
A_22_ 3’ O-Me GTP	0.45	+	0.38

^a^ IC_50_ were determined from the dose response inhibition of the GTase activity as indicated in Figure 2B and 2C for A_3_.

^b^ The formation of the covalent complex was determined as indicated in Figure 2D for each nucleotide analogue.

^c^ The efficiency of RNA capping was calculated by quantifying the nuclease P1 and alkaline phosphatase digestion resistant products formed for GTP and each nucleotide analogue as indicated in Figure 2F and normalizing it onto the value obtained with GTP.

^d^ RTP stands for Ribose triphosphate

^e^ The 1^st^ and 2^nd^ steps imply measuring the capping efficiency when RNA was incubated with the GTase and a purine analogue simultaneously, without prior isolation of the intermediate complex.

## Results

### Formation of a covalent intermediate with nucleotide analogues

In order to probe into the structural flexibility of the active site of a GTase, we tested the relative propensity of the PBCV-1 GTase to accommodate modified substrates through the use of nucleotide analogues. The nucleotide analogues harbored various modifications on both the ribose and the guanine base of GTP (Figure 1C).

**Figure 2:**
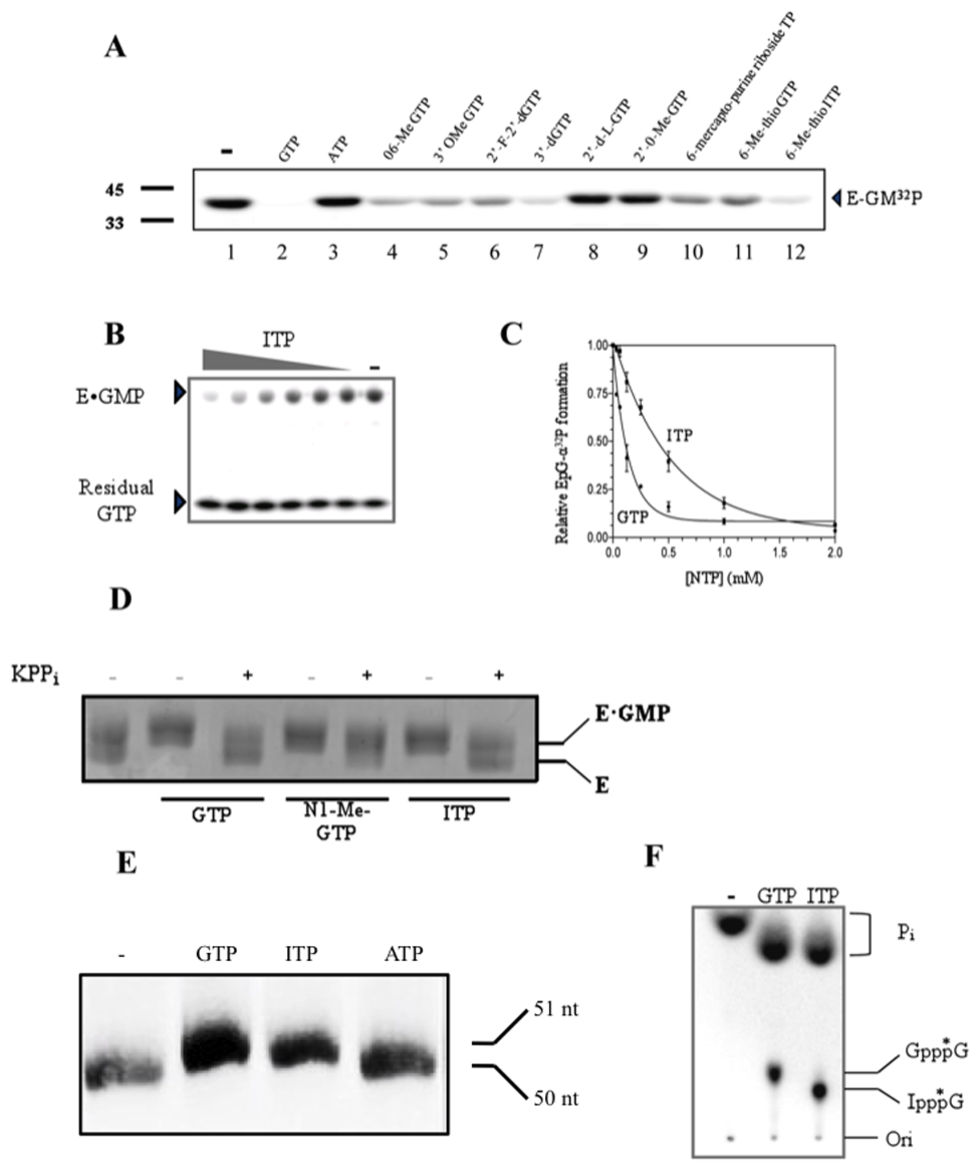
Biosynthesis of novel RNA cap structures (A) The PBCV-1 GTase was incubated with [α-^32^P] GTP in the absence or presence of unlabelled GTP (2 mM) (lanes 1 and 2) or purine analogues (2 mM) (lanes 3-12). An autoradiogram of the SDS-PAGE gel is shown. The location of the EpG complex is indicated on the right. (B) Competitive inhibition of the EpG complex formation by ITP (A_2_). Increasing concentrations of A_2_ (0.0625, 0.125, 0.25, 0.5, 1.0, 2.0 mM) were added to the standard GTase reaction containing [α-^32^P] GTP. An autoradiogram of the SDS-PAGE gel is shown. The location of the EpG complex is indicated on the left. (C) Dose-response inhibition of the PBCV-1 GTase by unlabelled GTP and ITP (A_2_). (D) Formation of the enzyme covalent intermediate. The PBCV-1 GTase was incubated with GTP (lanes 2 and 3) or nucleotide analogues (lanes 4 to 7) in the presence of either yeast pyrophosphatase or potassium pyrophosphate (5 mM). A Coomassie blue stain of the SDS-PAGE gel is shown. (E) Unlabelled GTP or nucleotide analogue (2 mM) was incubated with a ^32^P-radiolabeled RNA substrate of 50 nt in the presence of the PBCV-1 GTase and RTase. The RNA samples were extracted with phenol/chloroform, recovered by ethanol precipitation, and analyzed on a 20% UREA-PAGE. An autoradiogram of the gel is shown. The position of the unblocked RNA of 50 nt is indicated. (F) The RNA capping reaction was carried out with a 50 nt long 5’ terminally labelled RNA on the α phosphate. The reaction mixtures were heated for 5 min at 70°C, adjusted to 50 mM NaOAc, pH 5.2, and subjected to digestion by nuclease P1 for 60 min at 37°C and then adjusted to 50 mM Tris-HCl, pH 8, and digested by an alkaline phosphatase. The reaction products were analyzed by thin layer chromatography on a PEI-cellulose plate developed with 0.5 M LiCl and 1M formic acid. An autoradiogram of the plate is shown. The positions of the chromatographic origin (ori), GpppG, and of inorganic phosphate (P_i_) are indicated. P_i_ in lane 1 (negative control) migrates higher because this reaction was done in the absence of any RNA capping proteins. (*) indicates the position of the radiolabelled phosphate in the capped dinucleotide.

We initially monitored the ability of a library of 22 synthetic nucleotide analogues to inhibit the PBCV-1 GTase. The first step of the GTase reaction entails the nucleophilic attack of the α-phosphate of GTP by the enzyme and the subsequent formation of a covalent enzyme-GMP intermediate. The ability of the purified PBCV-1 GTase protein to form a covalent GMP-enzyme intermediate was detected by label transfer from [α-^32^P] GTP to the enzyme. A single SDS-stable GMP-enzyme complex that migrated as a 39.9 kDa species was detected following SDS-PAGE (Figure 2A, lane 1). In order to identify nucleotide analogues which could efficiently substitute GTP in the reaction under steady-state conditions, the candidate molecules (2 mM) were incubated in the presence of [α-^32^P] GTP and the PBCV-1 capping enzyme (10 µM). The presence of non-labelled GTP (2 mM) led to a maximum loss of the radiolabelled EpG signal (Figure 2A, lane 2) whereas ATP at the same concentration had no apparent effect (Figure 2A, lane 3). Each synthetic analogue inhibited the intermediate formation to a different extent (Figure 2A, lanes 4-12). The IC_50_ of the nucleotide analogues were then determined (Table 1). Increasing concentrations of non-labelled GTP or nucleotide analogue were added to the standard GTase reaction. An example, using ITP (A_2_) as an inhibitor is shown in Figure 2B and 2C. IC_50_ values ranged from 100 µM (high inhibition e.g. GTP) to 2.0 mM or more (low inhibition e.g. 2’dGTP). The fact that some nucleotide analogues led to inhibition of the PBCV-1 GTase activity at high concentrations (> 2.0 mM) raised the possibility that they might be acting as chelator for the essential divalent metal ion co-factor, and therefore these analogues were not included in subsequent analyses.

The ability of the GTase to directly use the nucleotide analogues as substrates was then assessed by incubating the PBCV-1 GTase with magnesium ions (5 mM) and the nucleotide analogues (2 mM). The reaction products were then analyzed by SDS-PAGE followed by Coomassie Blue staining, in order to visualize the appearance of a slower migrating species corresponding to the protein covalently bound to a nucleotide (Figure 2D) [24]. Reactions performed in the presence of analogues A_1_, A_2_, A_5-10_, A_13-14_, and A_20-22_ all led to the appearance of a slower migrating species relative to the unbound protein. Pyrophosphate, the GTase reaction product, promoted the release of NTP through reversal of the reaction, thus indicating that the use of different nucleotide analogues did not alter the reaction reversibility (Figures 1A and 2D). Notably we observed that all identified inhibitors of the first step of the GTase reaction formed a covalent intermediate.

### Nucleotide analogues as RNA cap donors

Having confirmed that the PBCV-1 GTase can efficiently form covalent intermediates with several nucleotide analogues, we next determined whether these intermediates had conserved the ability to be transferred onto a 5’-diphosphate RNA. The PBCV-1 GTase was therefore incubated with the appropriate nucleotide analogue, a 5’-α terminally labelled RNA and the PBCV-1 RTase. The reaction products were digested by nuclease P1 and alkaline phosphatase and resolved by thin layer chromatography. The resolved chromatogram revealed the presence of a digestion resistant species corresponding to GpppG when GTP was added to the RNA capping reaction mixture, thus confirming the transfer of GMP onto an acceptor RNA (Figure 2F). The fraction of capped RNA over the total transcripts was estimated to be 0.35 ±0.05 when GTP was the cap donor. The covalent intermediates formed from various purine triphosphate analogues showed varying degrees of efficiency in their ability to act as cap donors (Table 1). However, two of the analogues tested (A_9_ and A_10_) were clearly not transferable onto RNA. We conclude that the formation of the covalent E-NMP intermediate does not necessarily imply the completion of the second step of the GTase reaction A second assay was performed to demonstrate the transfer of nucleotide analogues onto RNA. A purified ^32^P-internally labelled RNA was incubated with the PBCV-1 GTase, the PBCV-1 RTase, magnesium ions and each nucleotide analogue separately. The reaction products were analyzed by Urea-PAGE. The addition of GTP or a nucleotide triphosphate (A_2_) analogue led to the formation of a slower migrating species corresponding to the capped RNA (Figure 2E). ATP, on the other hand had no effect. This clearly confirmed that some nucleotide analogues can act as cap donors and be transferred onto RNA, whereas others cannot.

### Translation of differentially capped RNAs *in cellulo*

The importance of modified RNA caps on both *in cellulo* RNA levels and translation was next investigated. Several studies have demonstrated that the N7-methyl guanosine cap structure is essential for the process of cap-dependent translation, since the association of a functional translation initiation complex requires the prior interaction of the RNA cap structure with the eukaryotic initiation factor 4E (eIF4E), an evolutionarily conserved subunit of the heterotrimeric eIF4F initiation complex [6]. Previous studies have shown that ^m7^GTP and numerous GTP analogues harboring various modifications can directly bind to eIF4E [26]. Having generated several novel 5’ cap structures bearing new modifications on both the base and the ribose moieties, we investigated their effects on RNA metabolism. Since the presence of a methyl group on the capping nucleotide is essential for the binding to eIF4E, we first evaluated the capacity of each modified cap to act as a substrate for a RNA (guanine-N7) methyltrasnferase (MTase), by using both the human and the yeast MTases. Our results show that both MTases can methylate RNAs blocked with A_2_, A_6_, A_7_, A_13_, A_14_ and A_22_, albeit to different extents (Table S1). However, attempts to methylate an RNA blocked with A_1_ were unsuccessful.

**Figure 3:**
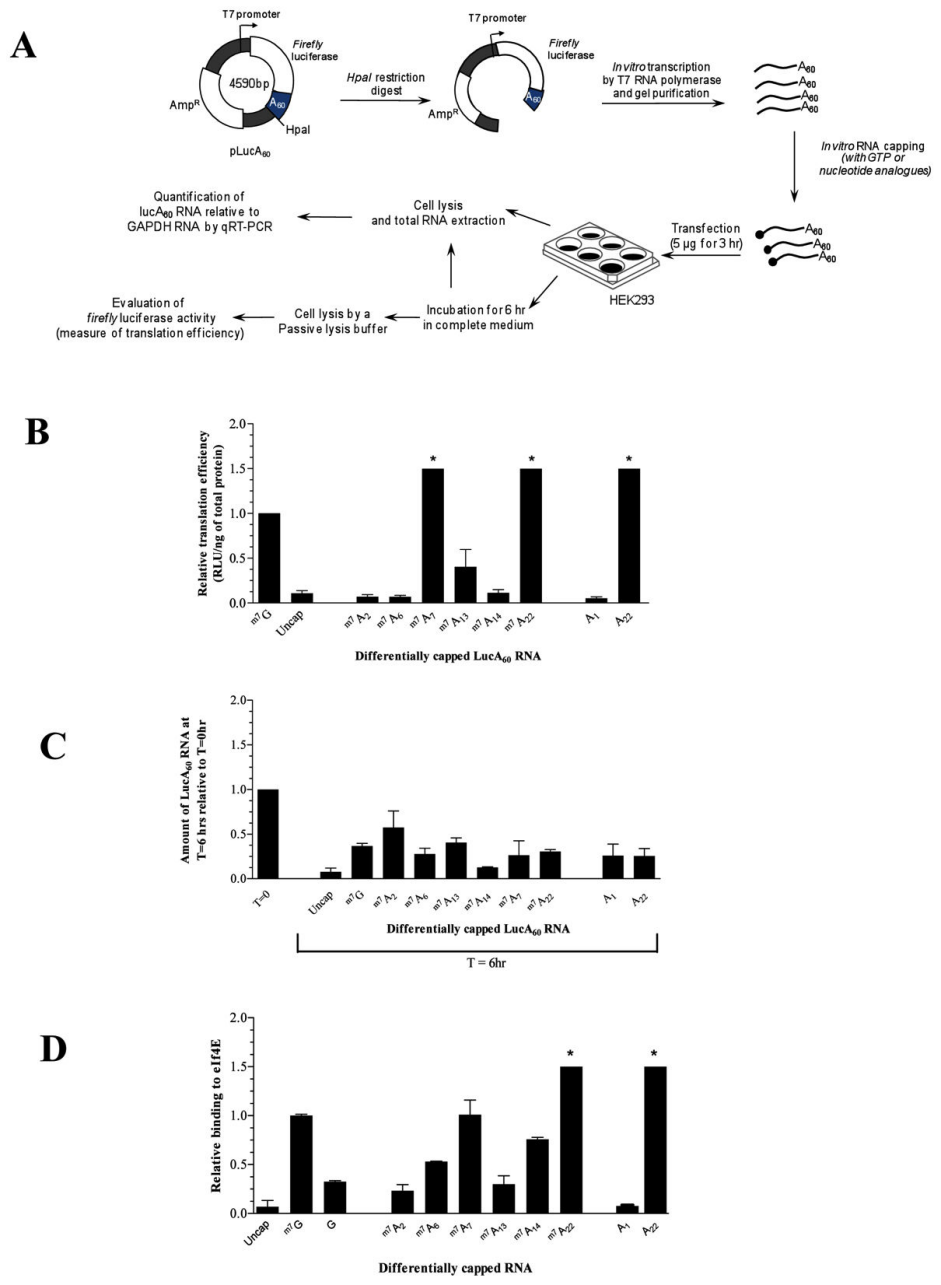
*In* *cellulo* and *in* *vitro* properties of the novel cap analogues (A) Schematic representation of the experimental procedure for the determination of the translation efficiency of differentially capped lucA_60_ RNA in HEK293 cells. (B) The relative translation efficiency was experimentally determined by quantifying *firefly* luciferase activity relative to the amount of total protein 6 hr post-transfection. Experimental data was adjusted relative to the capping efficiency (as determined in Table 1) of each analogue, and rationalized onto the ^m7^G cap. The error associated with each data set is less than ± 0.1. (*) indicates more than 1.5 fold difference relative to the translation efficiency of a naturally capped RNA. (C) The relative RNA level was evaluated by quantifying the amount of lucA60 RNA relative to the GAPDH RNA by qRT-PCR 0 hr and 6 hr post-transfection. (D) Binding to eIF4E was determined by fluorescence spectroscopy with a 30 nt long differentially capped RNA molecule. (*) indicates more than 1.5 fold difference relative to the binding observed for the natural ^m7^G capped RNA.

The ability of RNAs capped with nucleotide analogues and methylated at the N7 position to be translated was next investigated *in cellulo*. *Firefly* luciferase RNA (5 µg) harboring modified caps at their 5’ ends were transfected into HEK293 cells. An outline of the experimental procedure is shown in Figure 3A. Importantly, each transfection was performed in parallel with a positive ( ^m7^G cap) and a negative (uncap) control RNA. Luc A_60_ RNA levels relative to the GAPDH RNA level (normalizing control) were evaluated just after transfection and 6 hr post-transfection by qRT-PCR using specific primers. On account of the stress imposed on the cells by such chemical transfection procedures, earlier time points were discarded. As shown previously, a time frame between 5-24 hour periods was sufficient to detect differences in translation efficiency [25,26]. A 6 hr time point was chosen following a preliminary time course analysis which showed sufficient cell recovery and indicated maximum luciferase activity 6 hours post-transfection using the Qiagen Transmessenger reagent. The results were normalized onto the relative amount of lucA_60_ RNA possessing a natural cap (Figure 3B).

For each transfection assay, the luciferase activity of total cell extracts was also determined and corrected for the capping efficiency of each nucleotide analogue, and translation efficiency was determined relative to the positive control for each assay (Figure 3B). Interestingly, the translation efficiencies of the RNAs investigated did not reflect their relative efficiency as methyl acceptors. RNA capped with 3’ O-Me GTP (A_22_), despite its poor propensity to be a N7-methyl acceptor, displayed higher translation efficiency as compared to RNAs capped with A_2_, A_13_ or A_14_. Our results are in agreement with previous studies which showed that RNAs possessing an N7-(3’ O) dimethyl guanosine cap structure ( ^m7^A_22_) are translationally more active than RNAs possessing the natural cap [26-28].

**Figure 4:**
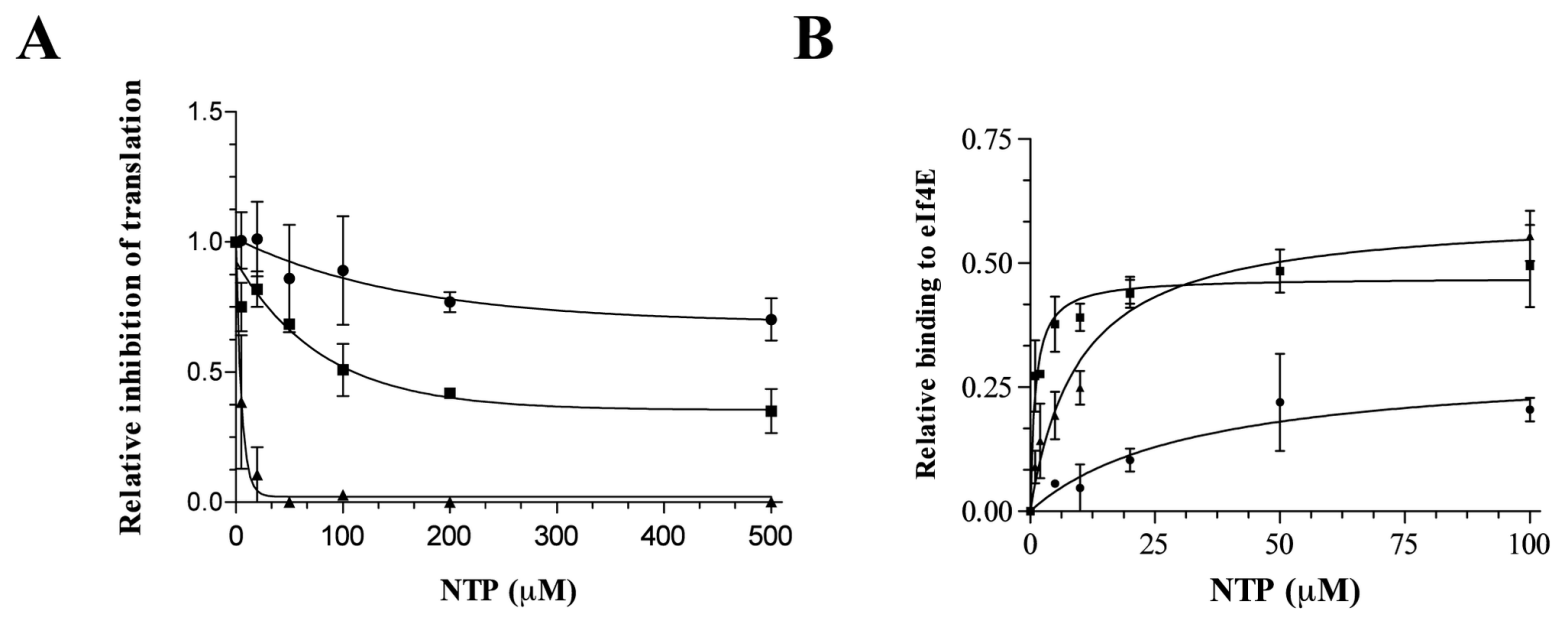
*In* *vitro* characterization of 3’ O-methyl GTP (A_22_) as an inhibitor of translation and as a binding partner to eIF4E (A) To the rabbit reticulocyte lysate (Promega) *in* *vitro* translation system, ^m7^G capped lucA_60_ RNA (1µg) and increasing amounts of NTP was added. Luciferase activity was measured after 10 minutes and plotted relative to the activity in the absence of any nucleotides. (B) Increasing amounts of NTP were added to a 2 µM solution of the purified protein in a binding buffer (50 mM Tris/HCl, pH 8.0, and 50 mM KOAc) and following excitation of tryptophan residues at 290 nm the emission spectrum was scanned from 310 to 440 nm. A saturation isotherm was generated from these data by plotting the change in fluorescence intensity at 333 nm as a function of added NTP. The data was fit to a binding curve. (■) indicates ^m7^GTP; (▲) indicates 3’ O methyl GTP (A_22_); and (●) represents GTP.

However, most intriguingly, the absence of the N7-methyl group (A_22_ cap) does not impact translation efficiency negatively. The translation efficiency of A_22_ and ^m7^A_22_ remain similar (within experimental error) despite the absence of the methyl group at the N7 position and consequently of the positive charge on the cap structure. The effect on translation of the 3’ O-methyl modification was further investigated by assessing the ability of 3’ O-methyl GTP (A_22_) to inhibit cap-dependent translation *in vitro*. LucA_60_ RNA possessing a standard N7-methyl guanosine cap structure was incubated with increasing amounts of either GTP, ^m7^GTP or A_22_ in the rabbit reticulocyte lysate translation system (Promega). Our results indicate that A_22_ leads to a rapid decrease in the translation of N7-methyl guanosine capped lucA_60_ RNA as compared to GTP (Figure 4A). Taking into consideration our *in cellulo* data, whereby an N7-methyl deficient A_22_ capped lucA_60_ RNA is translationally active; as well as the *in vitro* data which indicates that A_22_ can strongly inhibit the translation of an N7-methyl capped RNA, we concluded that in the context of cap-dependent translation, the presence or absence of the N7-methyl modification becomes secondary when alternative modifications are present on the capped residue.

### *In vitro* binding to eIF4E

In an effort to further analyze the translation profile obtained, we set out to determine the binding affinity of each novel RNA cap structure obtained to the eIF4E protein. The eIF4E protein harbors eight conserved tryptophan residues within its cap binding slot [29,30]. Therefore, binding affinity was evaluated by monitoring the quenching of the intrinsic fluorescence of the protein when incubated with a 30 nt long RNA possessing a natural or modified cap structure (Figure 3D). Our results echo previous studies in that for cap-dependent translation to occur, binding to eIF4E is a fundamental requirement. The affinity of eIF4E to an A_22_ capped RNA relative to a naturally capped RNA was more than 1.5 fold higher, in spite of the lack of the N7-methyl group on this cap analogue. This relates directly with the higher translation profile obtained for the A_22_ capped lucA_60_ RNA. Overall, these results indicate that cap-dependent translation can be sustained in the absence of the N7 modification of the RNA cap structure, provided that alternative modifications enable proper binding to the eIF4E protein. Following this result, we monitored the binding of 3’ O-methyl GTP (A_22_) directly to the purified eIF4E protein (Figure 4B). A similar *Kd* within the low micromolar range was obtained for both ^m7^GTP and 3’ O-methyl GTP whereas a much higher apparent binding constant was obtained for GTP (Figure 4B). Moreover, the observation that RNAs capped with A22 have stronger affinity to eIF4E than RNAs capped with m7GTP (Figure 3D) whereas free m7GTP binds more strongly than A22 to purified eIF4E (Figure 4B) strongly suggests that molecular determinants present in RNA also contribute to the binding to eIF4E. We therefore conclude that eIF4E can effectively bind to an N7-methyl deficient 3’ O-methyl guanosine cap structure.

## Discussion

RNA cap analogues are important biological tools for the study of RNA metabolism, and could prove to be potent novel therapeutic agents. We report that the active site of the model PBCV-1 GTase can accommodate several unnatural substrates, some of which can effectively be transferred onto acceptor RNA to form RNA cap analogues. Through the relative capacity of several nucleotide analogues to sustain each step of the GTase reaction, key insights into substrate recognition for RNA cap formation were revealed. For instance, our experimental results with regards to ITP (A_2_), which could efficiently substitute GTP in both steps of the GTase reaction, indicate a lack of importance of the exocyclic C2 amino group in substrate binding and catalysis (Table 1). Moreover, our results show that a hydroxyl group at the C8 position leads to complete loss of inhibition by the nucleotide analogue (A_12_, IC_50_ > 2.0 mM), whereas bromo or iodo groups at the same position rescues inhibition (A_13_, IC_50_=1.5 mM and A_14_, IC_50_=0.42 mM respectively) (Table 1). This is suggestive of an influence of the relative electronegativity of each substitution (χ of I < Br < O) on the guanine base. Taking into account that N7 modifiers (A_15_ and A_16_) completely abrogated GTase activity, we hypothesized that inductive effect due to increasingly electronegative substituents at the C8 position is affecting the hydrogen bond acceptor properties of the N7 position. In contrast to GTP, the lone pair of electrons at the N7 position in ATP is less available for interactions due to the basic chemical properties of the base [31]. Our results indicate that both N7 and O6 likely mediate interactions responsible for cap donor specificity.

### O6 modified analogues

It has previously been speculated from crystallographic data that GTP specificity of capping enzymes is mediated by the interactions of the O6 of GTP via a hydrogen bond with a conserved lysine residue (Lys188 in the PBCV-1 GTase) (Figure 1B) [9,32]. A sequence alignment between RNA capping enzymes tentatively places this conserved lysine residue in a motif (denoted IIIc), which ostensibly seems to be absent among DNA ligases (Figure S1). Interestingly, while alanine mutation of this lysine residue abolishes GTase activity, binding of GTP remains unaffected (Figure S3). Therefore, it is presumed that other amino acids are contributing to GTP binding. Nucleotide analogues harboring modifications at the O6 position were used to investigate this issue. With the exception of 6-thio ITP (A_9_) (IC_50_=1.7 mM) and 6-Me-thio ITP (A_10_) (IC_50_=1.2 mM), O6 modified analogues that were tested did not differ significantly relative to GTP (IC_50_=0.1 mM) in their ability to inhibit the first step of the reaction (Table 1-column 2). The similar IC_50_ of A_5_, A_6_ and A_7_ indicate that the extent of Lys188 interactions with nucleotide analogues bearing C6 modifiers is permeable to steric constraints and variations in the electrostatic potential. However, in all cases, the transfer onto an acceptor RNA was heavily compromised during the second step of the GTase reaction O6-Me GTP (A_7_) was transferred ~50% as efficiently as GTP onto an RNA, whereas A_5_ and A_6_ were poor cap donors (8% and 16% respectively relative to GTP) (Table 1-column 4). Our results indicate that along with Lys188 and O6 hydrogen bonding, it is the architecture of this region of the active site which may be mediating substrate binding and contributing to the reaction progress. In addition, given that only O6-Me GMP (A_7_) retains appreciable transferability onto RNA, we suggest that only oxygen at the C6 position could preserve the subtleties of interactions with Lys188, thereby allowing accurate substrate alignment for the intermediate complex formation while also retaining the transferability of the bound nucleotide analogue onto an acceptor RNA.

### 2’ and 3’ modified GTP analogues

Previous studies have shown that 2’dGTP (A_18_) cannot be hydrolyzed by the PBCV-1 GTase [24]. This is suggestive of an important role of the 2’OH group. This might be due to the following reasons: (i) the 3’-*endo* conformation, which is preferred by ribonucleotides but not deoxyribonucleotides may be important; (ii) the inductive effect of the 2’OH group may be important; (iii) the 2’OH group coordinates a critical metal ion or (iv) binding of the 2’OH may be a pre-requisite for proper alignment of the triphosphate moiety.

With the exception of the *poxvirus* capping enzyme, exemplified by the vaccinia virus D1 protein, most known capping enzymes are unable to hydrolyze 2’dGTP [33,34]. Since the D1 protein shares the same conserved motifs as the PBCV-1 GTase, it implies that the 2’OH must have a preponderant role in ligand binding rather than catalysis. Therefore, the hypothesis that the 2’OH may be participating in the coordination of a critical metal ion is unlikely. The sugar moiety of 2’F-2’dGTP (A_20_) has a net preference for the 3’-*endo* conformation and the 2’F substituent has a stronger inductive effect than the hydroxyl group in GTP [35]. The fact that the 2’F substituent negatively affects the first step of the GTase reaction by drastically increasing the IC_50_ (1.4 mM for A_20_ compared to 0.10 mM for GTP) is a clear indication that neither the preference for the 3’-*endo* conformation nor the inductive effect explains the preference of the PBCV-1 GTase for GTP over 2’dGTP. Thus far, our results indicate that the 2’OH is involved in ligand positioning, without necessarily being involved in catalysis. From, previous structure-function analysis in the budding yeast and the mouse capping enzymes, the 2’OH group has been inferred to be interacting with an essential Motif III glutamate –Glu131 in the PBCV-1 GTase, with which it forms a hydrogen bond (Figure 1B and Figure S1) [36]. The pH dependency of the inhibition of the first step of the GTase reaction by 2’O-Me GTP (A_19_) further substantiated this conclusion. A_19_ does not inhibit the GTase reaction at pH 7.5 (Table 1). However, decreasing the pH reduced the ease of formation of the radiolabelled covalent EpG intermediate, thereby indicating that at lower pH, A_19_ can effectively inhibit the reaction (Figure S2). Decreasing pH infers protonation of basic residues (like Glu131) which renders possible the otherwise unfavorable interaction between the glutamate residue and the 2’ oxygen of 2’O-Me GTP at a pH of 7.5. Likewise, 2’F-2’dGTP also shows a pH dependency for the first step of the reaction, albeit to a lesser extent than 2’O-Me GTP. We conclude that the essentiality of the 2’OH group in RNA capping resides mainly in proper ligand positioning.

In contrast to 2’ dGTP, the absence of the 3’ OH group (A_21_) did not affect the first step of the reaction, while its substitution with 3’ O-Me (A_22_) led to an increase in the IC_50_; a change that we attributed to steric hindrance. However, RNA capping with 3’ dGTP was markedly inferior relative to 3’ O-Me GTP (10% and ~40% respectively relative to GTP). Crystallographic data of the *C. albicans* GTase indicated the presence of a phosphate ligand in contact with the 3’ hydroxyl group of the bound GMP within the active site of the enzyme. Also taking into account previous structure function studies, our results corroborate this hypothesis by clearly demonstrating that the role of the 3’ OH group lies mainly in the second step of the GTase reaction [32,37].

### *In cellulo* properties of artificial cap structures

The subset of RNAs generated with modified cap structures at their 5’ ends were evaluated for their translational properties in HEK293 cells. Regarding the relative level of RNAs capped with various analogues 6 hours post-transfection, our results were consistent with the fact that the presence of a blocking residue at the 5’ end of an RNA was sufficient to protect it from rapid degradation as compared to an uncapped RNA (Figure 3C) [10]. Previous studies addressing the low ability of ^m7^IMP and ^m7^IDP to inhibit translation have demonstrated their poor binding affinity for the mammalian eIF4E, and in agreement, a very low translation efficiency was observed for a N7-methyl inosine ( ^m7^A_2_) capped RNA [38,39]. In addition, our results concerning an N7-(3’ O)-dimethyl guanosine ( ^m7^A_22_) capped RNA are consistent with previous observations that an RNA capped with ^m7^A_22_ is translationally more active than RNAs possessing the natural cap (Figure 3B) [26,28,40].

Interestingly, in spite of a poorer N7-methylation efficiency of the 3’ O-methyl guanosine (A_22_) cap structure as compared to the conventional guanosine cap (Table S1), the ^m7^A_22_ capped lucA_60_ RNA was efficiently translated *in cellulo*. Therefore, in order to look into the possible effects of alternative methylations on the capped guanosine residue, we monitored the translation efficiency of the N7-methyl deficient A_22_ capped lucA_60_ RNA as well as the N7-methyl deficient A_1_ capped RNA. The A_1_ capped RNA has been shown to be completely inert to N7-methylation by RNA (guanine-N7) methyltransferases (Table S1). While the poor translation efficiency of the A_1_ capped RNA was consistent with the absence of the N7-methyl group, the much higher translation efficiency of the A_22_ capped lucA_60_ RNA, despite the absence of the N7-methyl group, was totally unexpected. It can be argued that post-transfection the A_22_ capped LucA_60_ RNA can be methylated intracellularly at the N7 position. However, our *in vitro* N7-methylation data indicates that N7-methylation of A_22_ capped LucA_60_ is very poor. This suggests that most A_22_ capped LucA_60_ would remain N7-methyl deficient after transfection. To the best of our knowledge, this is the first report of an N7-methyl deficient capped RNA which can support cap-dependent translation *in cellulo*. Previous reports have evaluated the importance of the N7-methyl addition solely in the context of the natural N7-methyl guanosine cap. Compensation for the absence of the N7-methyl group by alternative modifications on the cap structure had not been looked into before. Our in vitro binding studies indicate that the relative binding affinity of eIF4E to the modified cap structure was sufficient to explain the observed translation efficiency. While a GpppG capped RNA is not bound by eIF4E, the presence of a 3’ O-methyl group (A_22_) restores eIF4E binding, and translation (Figure 3B, 3D and 4B). The major implication of this result is that cap binding by eIF4E does not necessarily require a positively charged capped nucleotide, as is the case for the natural RNA cap structure. Crystallographic data on eIF4E indicates that the cap binding slot is essentially divided into a positively charged region which binds the phosphate bridge, and a hydrophobic region where the charged N7-methyl guanosine cap is stacked between two conserved tryptophan residues through cation-π-π interactions [41]. The N7-methyl group is involved in Van der Waal’s interactions only, and has been shown to be substitutable with various alkyl groups without being deleterious to eIF4E binding [42]. It was therefore proposed that the main purpose of the N7-methyl group is to confer a positive charge to the cap. Because most previous studies used the premise that cap binding to the eIF4E protein requires a positively charged RNA cap analogue, the necessity of the N7-methyl group in the context of other modifications on the RNA cap structure had not been previously addressed. The ribose moiety of the capped guanosine residue has not been described to be involved in cap binding by eIF4E. In fact, the stacking interactions of the base, and interactions between the phosphate chain and positively charged amino acids are the main energetic contributions for cap binding to eIF4E [43,44]. It is postulated that the phosphate bridge of the RNA cap acts as an anchor to enable the capped N7-guanosine to interact within the cap-binding slot. Our observation that RNA blocked with 3’ O-methyl guanosine (A_22_) could be efficiently bound by eIF4E was therefore very intriguing. In view of our results relative to the A_22_ cap structure, we conclude that other factors are necessarily rendering the interactions of A_22_ with eIF4E favorable (Figure 4B).

The importance of the N7-methyl group of the cap structure has been highlighted in several studies. Our results indicate that cap-dependent translation can occur in the absence of the N7-methyl group on the cap structure provided that alternative modifications enable appropriate binding to eIF4E. The use of more novel RNA cap analogues would most certainly provide more insights into this issue. Finally since eIF4E is over-expressed in various cancers, and is under investigation as a potential drug target, our findings also have major implications for the design and synthesis of potential therapeutic agents targeting the eIF4E protein [45-47].

## Supporting Information

Figure S1 **Structural conservation in GTases and ligases.** The amino acid sequences of GTases from *Paramecium* *bursaria* Chlorella virus*-*1 (Chv), *S. cerevisiae* (Sce), *S.* pombe (Spo) and *C. albicans* (Cal) are aligned with ligases from the T7 phage (T7), Vaccinia virus (Vac) and Sce (*S. cerevisiae*).
(JPG)

Figure S2 **pH dependency of the inhibition by 2** ’ **modified nucleotide analogues**. (A) pH dependency for the inhibition by A_3_. The PBCV-1 GTase was incubated with [α-^32^P] GTP in the presence of either GTP (0.5 mM) or A_19_ or A_20_ (0.5 mM) in a standard GTase buffer ranging from a pH of 5 to 9. The reactions were resolved by SDS-PAGE and analyzed by a Phosphorimager. The formation of the radiolabelled E-GMP complex was quantified and its relative ease of formation is plotted as a function of the pH. High ease of formation implies low inhibition by either GTP or the nucleotide analogue, while low ease of formation implies high inhibition by the unlabelled nucleotides.
(TIF)

Figure S3 **Binding of GTP to the wild-type and K188A mutant of the PBCV-1 GTase.** Increasing amounts of GTP were added to a 2 µM solution of the enzyme in binding buffer (50 mM Tris/HCl, pH 8.0, and 50 mM KOAc) and the emission spectrum was scanned from 310 to 440 nm, following excitation of tryptophan residues at 290 nm. (●) indicates wild-type enzyme and (▲) indicates the K188A mutant enzyme.
(TIF)

Table S1 **RNA (guanine-N7) methyltransferase activities with RNA capped with nucleotide analogues.**
(TIF)
