# Supplementary figures and images for: Enzymatic Synthesis of RNAs Capped with Nucleotide Analogues Reveals the Molecular Basis for Substrate Selectivity of RNA Capping Enzyme: Impacts on RNA Metabolism
Source: PLoS One. 2013 Sep 25;8(9):e75310. doi: 10.1371/journal.pone.0075310 (PMC3783409; doi:10.1371/journal.pone.0075310)

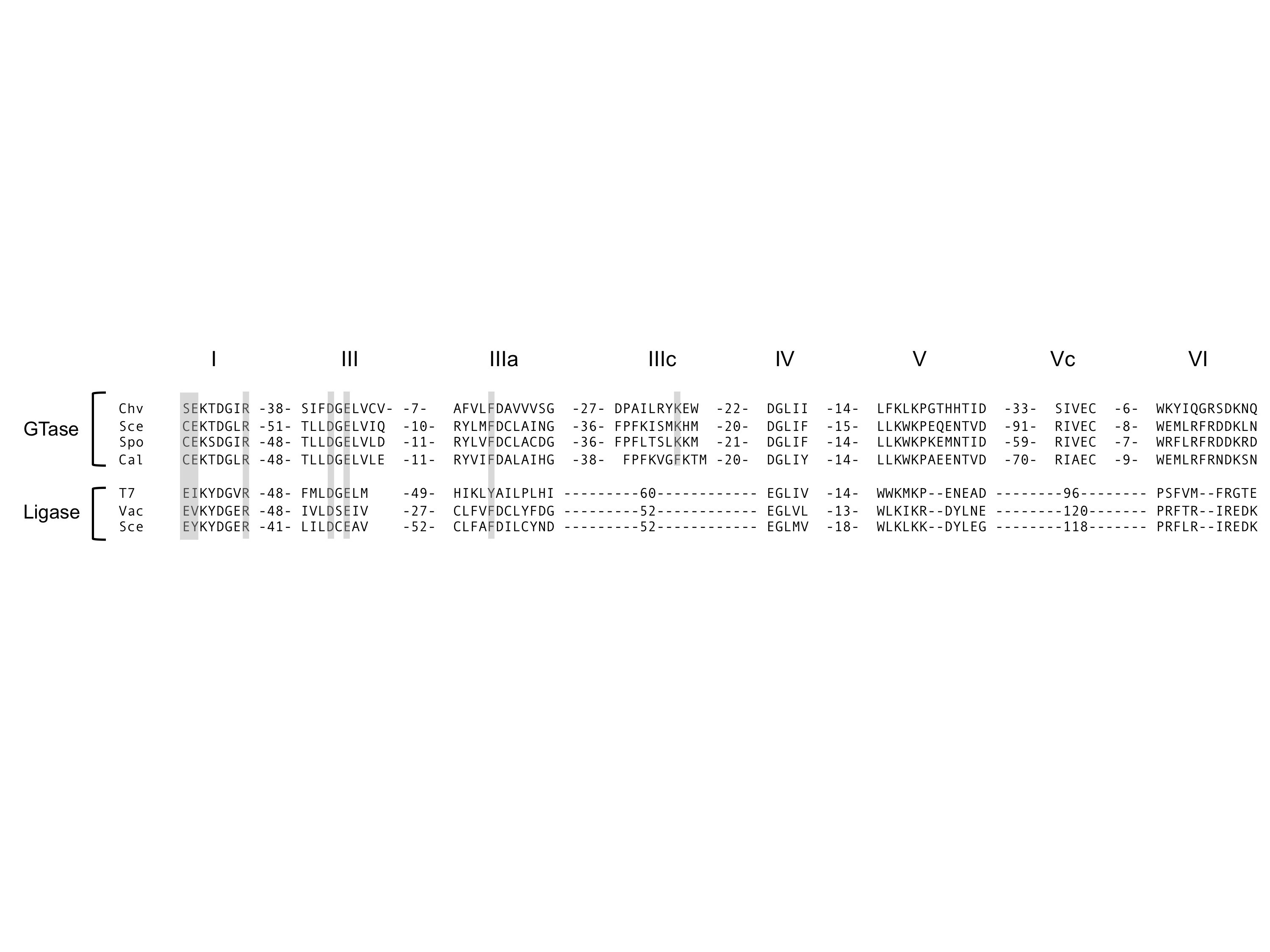

Supplement: Figure S1 — Structural conservation in GTases and ligases. The amino acid sequences of GTases from Paramecium bursaria Chlorella virus-1 (Chv), S. cerevisiae (Sce), S. pombe (Spo) and C. albicans (Cal) are aligned with ligases from the T7 phage (T7), Vaccinia virus (Vac) and Sce (S. cerevisiae). (JPG) [file pone.0075310.s001.jpg]

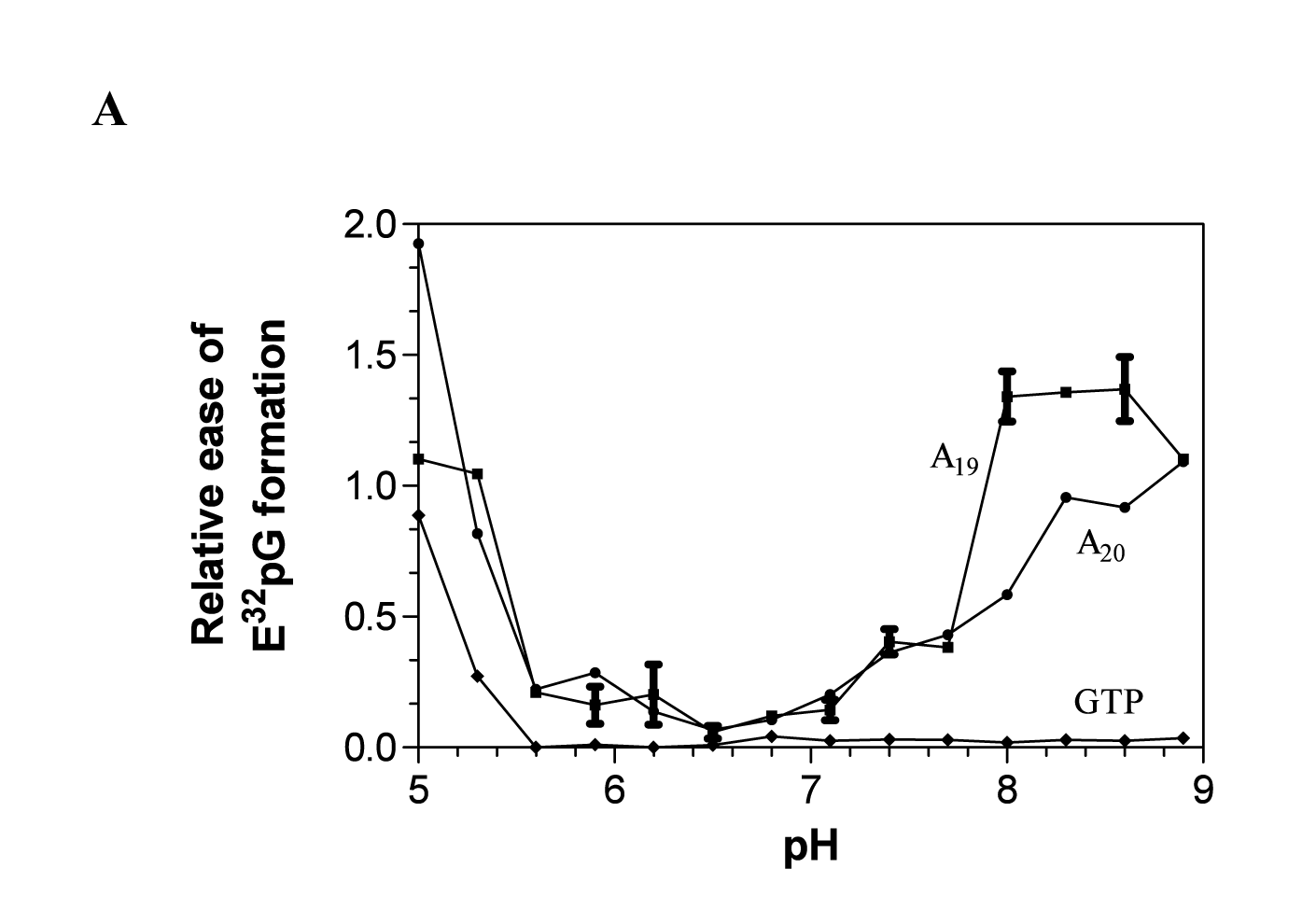

Supplement: Figure S2 — pH dependency of the inhibition by 2 ’ modified nucleotide analogues. (A) pH dependency for the inhibition by A3. The PBCV-1 GTase was incubated with [α-32P] GTP in the presence of either GTP (0.5 mM) or A19 or A20 (0.5 mM) in a standard GTase buffer ranging from a pH of 5 to 9. The reactions were resolved by SDS-PAGE and analyzed by a Phosphorimager. The formation of the radiolabelled E-GMP complex was quantified and its relative ease of formation is plotted as a function of the pH. High ease of formation implies low inhibition by either GTP or the nucleotide analogue, while low ease of formation implies high inhibition by the unlabelled nucleotides. (TIF) [file pone.0075310.s002.tif]

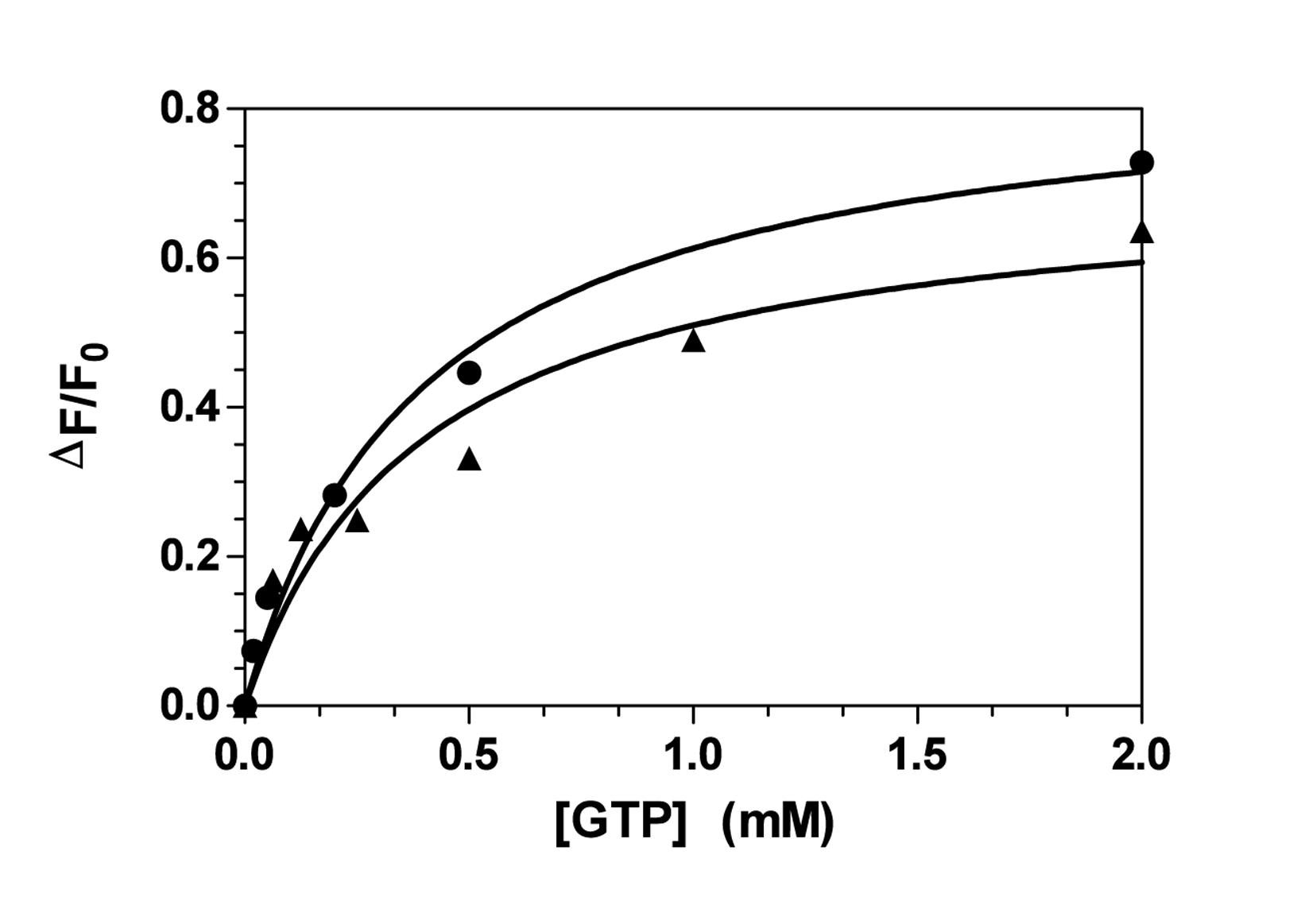

Supplement: Figure S3 — Binding of GTP to the wild-type and K188A mutant of the PBCV-1 GTase. Increasing amounts of GTP were added to a 2 µM solution of the enzyme in binding buffer (50 mM Tris/HCl, pH 8.0, and 50 mM KOAc) and the emission spectrum was scanned from 310 to 440 nm, following excitation of tryptophan residues at 290 nm. (●) indicates wild-type enzyme and (▲) indicates the K188A mutant enzyme. (TIF) [file pone.0075310.s003.tif]

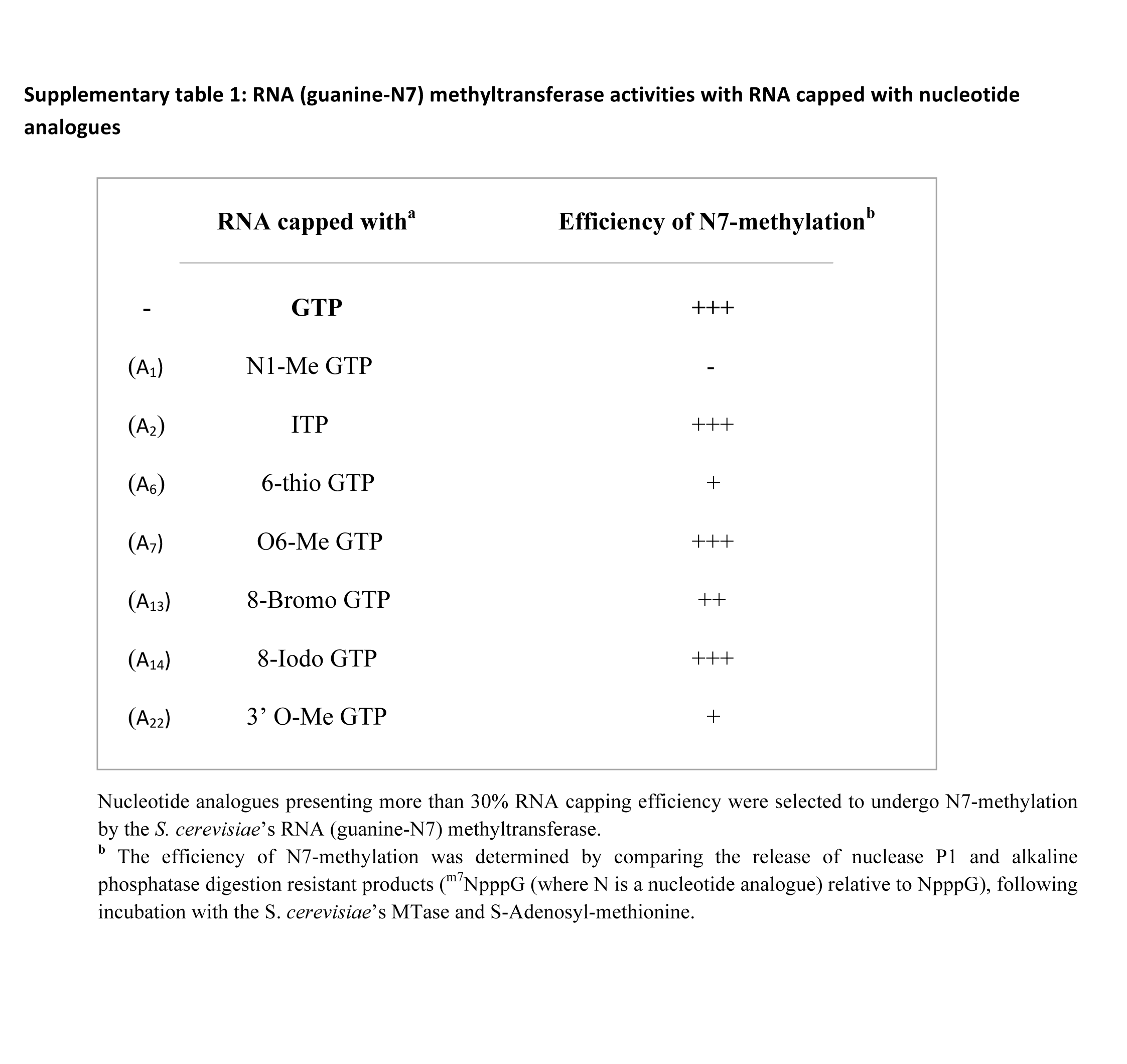

Supplement: Table S1 — RNA (guanine-N7) methyltransferase activities with RNA capped with nucleotide analogues. (TIF) [file pone.0075310.s004.tif]
